# A Feasibility Study on a Telemedicine Hybrid Protocol for Preoperative Anesthetic Assessment

**DOI:** 10.7759/cureus.40449

**Published:** 2023-06-15

**Authors:** Jia Xin Chai, Wan Yen Lim, Angie Phui Sze Au Yong, Sharon Gek Kim Ong

**Affiliations:** 1 Department of Anesthesiology, Sengkang General Hospital, Singapore, SGP; 2 Division of Anaesthesiology and Perioperative Sciences, Singapore General Hospital, Singapore, SGP

**Keywords:** telemedicine utilization, telemedicine experience, video telemedicine, telemedicine (tm), telemedicine patient satisfaction, patient satisfaction with telehealth, telehealth, preoperative assessment and risk management, perioperative medicine, video consultation

## Abstract

Background

Over the past decade, telemedicine has experienced significant growth due to technological advancement, and the coronavirus disease 2019 (COVID-19) pandemic further accelerated its adoption. However, the field of anesthesiology has been slow in integrating and embracing telemedicine compared to other medical specialties.

Methods

We conducted an observational pilot feasibility study at a tertiary hospital in Singapore to assess the viability of a telemedicine hybrid protocol for preoperative anesthetic assessment. The study included patients aged 21 to 65 years, classified as American Society of Anesthesiology (ASA) physical status class 1 or 2, with a body mass index (BMI) below 35 kg/m^2^, who were capable of managing video conferencing. The patients selected were scheduled for low-risk surgeries. The primary objective was to evaluate the medical and technical feasibility of our telemedicine hybrid protocol, while the secondary objectives included assessing patient satisfaction and obtaining feedback from relevant stakeholders.

Results

From November 2021 to April 2022, a total of 116 patients were recruited, with 96 patients completing the study. No technical difficulties, surgical case cancellations, or incidents of unanticipated difficult airways were reported. The majority of survey respondents (88%) expressed satisfaction with the video consultation and indicated a preference for it over physical consultations for future preoperative anesthesia evaluations.

Conclusion

Based on our findings, a telemedicine hybrid protocol for preoperative anesthetic assessment demonstrated both technical and medical feasibility while yielding high patient satisfaction. Future research could focus on expanding the protocol to encompass more complex surgeries and include patients with higher ASA status.

## Introduction

Telemedicine has witnessed rapid growth in the past decade, driven by advancements in technology. The recent coronavirus disease 2019 (COVID-19) pandemic has further accelerated its popularity and utilization. Recognizing the potential of telemedicine in reducing virus transmission through its remote capabilities, the World Health Organization (WHO) has also issued guidance for its implementation [1].

The earliest documented use of telemedicine in anesthesiology can be traced back to a 2004 case series, where ten patients underwent preoperative evaluation using a remote facility equipped with cameras [2]. Both patients and anesthesiologists reported high satisfaction scores. Subsequent studies on virtual anesthesia preoperative evaluation have yielded similar positive results [3]. Furthermore, in cases where patients initially underwent a telemedicine consult and subsequently required additional testing or in-person consultations, the initial telemedicine consultation reduced the duration of subsequent physical consultations by an average of 24 minutes, leading to improved overall clinical efficiency. Additional advantages reported include the elimination of commute and waiting times, convenience, outreach to rural areas [4], and significant cost savings by reducing work absences and childcare needs. Notably, the rates of surgical case cancellations remained unchanged [5]. However, telemedicine also presents certain limitations, including technical difficulties, limited access to videoconferencing systems, and increased costs associated with equipment setup [6,7].

Despite the reported advantages of telemedicine, there were limited video consultation-based protocols for preoperative anesthesia evaluation published when this pilot study was conducted [8]. Numerous video consultation-based studies have since been published in the past two years [9-11]. These were implementation studies focusing on the feasibility of specific protocols. However, they encompassed different surgical populations across various geographical locations. While high success rates and patient satisfaction have been reported, these studies have also highlighted challenges unique to their respective centers and patient populations. Consequently, the implementation of a telemedicine protocol within our local context remains unknown. Therefore, we conducted an observational study with the primary objective of evaluating the medical and technical feasibility of our video consultation hybrid protocol for preoperative anesthesia evaluation. The secondary objectives included assessing patient satisfaction and obtaining feedback from relevant stakeholders such as surgeons, nurses, and clinic assistants.

## Materials and methods

Ethics approval was obtained from the Singhealth Centralised Institutional Research Board (CIRB Ref: 2021/2348) prior to the commencement of the study. A total of 100 patients were planned to be recruited at Sengkang General Hospital between July 2021 and April 2022. Since this was a pilot feasibility study, the sample size represented a convenience sample, and power calculations were not performed. Participation in the study was voluntary, and written informed consent was obtained from all participants before their inclusion.

The inclusion criteria comprised adult patients aged 21 to 65 years scheduled for elective surgery, classified as American Society of Anaesthesiology (ASA) physical status class 1 or 2, with a body mass index (BMI) of less than 35 kg/m^2^ or weight below 100 kg, and having the ability to participate in a video conference using an internet-enabled device. These criteria were selected to enhance patient safety and minimize the risk of surgical cancellations. Patients were excluded if they had a diagnosis of hearing or cognitive impairment, a history of anesthetic complications or difficult airway, or lacked a local contact number. Surgical criteria included surgeries that lasted less than four hours and estimated blood loss of less than 500 ml. The eligible surgeries are presented in Table 1.

**Table 1:** Eligible surgeries for video consultation recruitment

Surgical disciplines	Examples of eligible surgeries	
General Surgery	i. Laparoscopic or open hernia repair	
	ii. Uncomplicated cholecystectomy	
	iii. Examination under anesthesia, fistulectomy	
	iv. Haemorrhoidectomy	
	v. Superficial cysts excision	
	vi. Incision and drainage of abscesses	
	vii. Simple mastectomy (without flap or reconstruction), lumpectomy	
Orthopedics	i. Hand, elbow or shoulder surgery	
	ii. Knee replacement, meniscus, ligamentous surgery	
	iii. Foot and ankle surgery	
Otorhinolaryngology	i. Ear surgery e.g. myringoplasty, mastoidectomy, stapedectomy	
	ii. Tonsillectomy	
	iii. Septoplasty, functional endoscopic sinus surgery	
	iv. Parotidectomy, thyroidectomy	
Urology	i. Vasectomy	
	ii. Circumcision	
	iii. Cystoscopy	
	iv. MRI- guided prostate biopsy	
	v. Urethral stent insertion	
	vi. Transurethral resection of prostate, transurethral resection of bladder tumor	

**Figure 1:**
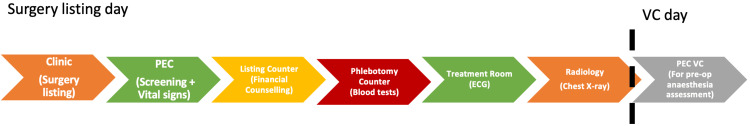
Patient journey for video consultation (VC) hybrid protocol

Eligible patients were initially identified during their surgical clinic visit and promptly referred to the anesthetic Preoperative Evaluation Clinic (PEC) for recruitment. Consent for recruitment was taken by a research coordinator at the PEC. Subsequently, a medical officer conducted a comprehensive physical examination focusing on the cardiorespiratory system, airway, and dentition. Additionally, vital parameters including blood pressure, pulse rate, and oxygen saturation were assessed. Routine preoperative investigations, such as blood tests, electrocardiograms, and chest X-rays, were also performed. The patient's journey from the initial recruitment to the video consultation appointment is illustrated in Figure 1.

We utilized the Zoom video conferencing platform (Zoom Video Communications Inc., San Jose, California), for conducting the video consultations. Eligible patients received appointment details through text messages and received a phone call reminder one day prior to their scheduled appointment. All hospital computers used for video consultations were equipped with the Zoom video conferencing software (version 5.2.0, 2020). To ensure data security, end-to-end encryption was implemented on password-protected computers connected to the hospital's secured networks. Furthermore, video recording functions were disabled to protect privacy. The details of the video consultations were documented in the electronic medical records by the attending doctor. Additionally, all doctors conducting video consultations had completed an online telemedicine accreditation module provided by the Ministry of Health, Singapore (Telemedicine: Use, Limitations, and Implementation Supplementary Material 1).

In accordance with national telemedicine guidelines, patients were required to provide proof of identity at the beginning of the video consultation [12]. Exact patient locations were also obtained to enable appropriate emergency service response if urgent care was needed. The anesthetic medical officer then obtained a relevant medical history and performed a modified virtual airway assessment using the upper lip bite test [13]. Patients were counseled on anesthetic risks after assessment, utilizing scoring tools such as STOP-Bang for obstructive sleep apnea screening [14] and Lee's Revised Cardiac Index [15] as per standard practice. Data on surgical case cancellations and adverse perioperative events, such as unanticipated difficult airways, were also collected.

Following the video consultation, patients were invited to complete an electronic patient satisfaction survey (see Appendix 1), which was adapted from validated patient experience questionnaires [16,17]. The survey link was sent via text message to their registered mobile numbers. The electronic survey was done on FormSG, an online form builder for public healthcare clusters for data collection (Open Government Product, Government Technology Agency, Singapore). Feedback regarding workflow and operational challenges was also solicited from referring surgeons, PEC medical officers, and administrative support staff. The obtained data was analysed using Microsoft Excel (Microsoft Corp, Redmond, WA, USA). Descriptive analysis was applied to calculate frequencies with corresponding proportions. 

## Results

Between November 2021 and April 2022, a total of 116 patients were enrolled in the study. Out of these, 96 patients successfully completed the video consultation, resulting in a completion rate of 82.8%. Among the 20 patients who dropped out, seven canceled their surgeries, seven had their surgeries postponed, five opted for physical consultations, and one withdrew from the study. Of the five conversions to physical consultations, three were due to surgery rescheduling affected by the COVID-19 pandemic, while the remaining two were due to patient preference and login platform issues. The median time from a video consultation appointment to surgery was 11.5 days, compared to 14 days for physical consultation to surgery.

Table 2 presents the demographic profile of the recruited patients. The majority (60%) of patients were aged between 30 to 49 years, and 56% were classified as ASA I. Around 88% of patients had a BMI of less than 30. The majority of patients were recruited from the Urology discipline (47%) and Orthopedics discipline (28%). Day Surgery (DS) and Same Day Admission (SDA) cases accounted for 95% of the surgeries.

**Table 2:** Patient demographics

Demographics		Number	Percentage (%)	Total
Age	20-29	19	20	96
	30-39	37	38	
	40-49	21	22	
	50-59	14	15	
	60-65	5	5	
ASA	1	54	56	96
	2	42	44	
BMI (kg/m^2^)	<25	54	56	96
	25 to 30	31	32	
	30 to 35	11	12	
Surgical Discipline	Otorhinolaryngology	5	5	96
	General Surgery	19	20	
	Orthopedic	27	28	
	Urology	45	47	
Type of Admission	Day Surgery	69	72	96
	Same Day Admission	22	23	
	Short Stay Ward (<24 hours stay)	5	5	

The type and distribution of procedures according to surgical disciplines are shown in Table 3. Within the Urology discipline, the majority of cases were simple day procedures such as vasectomies (51%) and circumcisions (29%). Hand and clavicle surgeries constituted 52% of orthopedic procedures, while knee arthroscopies accounted for 30%.

**Table 3:** Distribution of surgical procedures according to surgical disciplines

Surgical disciplines	Types of surgeries	Number	Percentage (%)	Total
Urology	Vasectomy	23	51	45
	Circumcision	13	29	
	Transurethral resection of the prostate	4	9	
	Others (transurethral resection of bladder tumor, cystolitholapaxy, laparoscopic Boari flap)	5	11	
Orthopedics	Shoulder, clavicle, hand surgery	14	52	27
	Knee surgery (athroscopy, unicompartmental arthroplasty)	8	30	
	Spine surgery (microdiscectomy)	3	11	
	Foot and ankle surgery (arthroscopy)	2	7	
General Surgery	Breast (Simple mastectomy, lumpectomy)	4	21	19
	Haemorrhoidectomy	10	53	
	Lump excision	1	5	
	Appendicectomy, cholecystectomy, closure of colostomy	4	21	
Otorhinolaryngology	Turbinoplasty, septoplasty	2	40	5
	Tonsillectomy, sinus surgery	2	40	
	Thyroid surgery (Hemithyroidectomy)	1	20	

The patient satisfaction survey results are presented in Table 4. Out of 96 patients, 79 responded (85% response rate). The majority of respondents (95%) agreed that the technical setup was easy, and 94% understood the explanations regarding anesthesia options. Nearly all respondents (98%) understood the preoperative instructions concerning fasting and medications. All respondents (100%) felt that the consultation duration was sufficient to address their concerns. Almost all respondents (99%) expressed satisfaction with the quality of service provided through the video consultation. Furthermore, 88% of respondents indicated a preference for video consultation over physical consultation for future preoperative anesthesia evaluations due to convenience, ease of use, and time saved on commuting and waiting. Minor technical issues, such as login or audio difficulties, were resolved without incident. Both patients and telemedicine providers found the technical setup and the Zoom platform easy to use.

**Table 4:** Patient satisfaction survey responses (n=79)

Question	Strongly agree	Agree	Neutral	Disagree	Strongly disagree
The technical setup for video consultation was easy for me	54	21	4	0	0
I understood the various anesthesia options that were explained to me over the video consultation.	44	30	4	1	0
I understood the preoperative instructions regarding fasting and medications during the video consultation.	51	26	1	0	0
I had time to ask questions during the video consultation.	56	23	0	0	0
My concerns were adequately addressed during the video consultation.	51	26	2	0	0
I found the duration of the video consultation to be adequate.	54	25	0	0	0
Overall, I am satisfied with the quality of service provided over the video consultation.	58	20	1	0	0
I would choose video consultation over physical consultation for any future preoperative anesthesia evaluation.	44	25	9	1	0

There were no surgery case cancellations for all patients who underwent video consultation. Of the 96 patients, 93 (96%) had upper lip bite class I and II, and there were no instances of difficult airways on the day of surgery. There was a good correlation between the virtual upper lip bite test with the physical airway assessment.

Stakeholders' feedback was positive. Surgeons expressed high satisfaction due to the ease of referral and increased availability of slots compared to physical appointments. Importantly, there were no surgical cancellations, which contributed to increased acceptance and referrals.

## Discussion

Our feasibility study demonstrated the successful implementation of a video consultation hybrid protocol, incorporating carefully selected patient and surgical criteria, which resulted in high patient satisfaction. To the best of our knowledge, this is the first study in the literature to examine a hybrid protocol for technical feasibility and patient outcomes. Other published preoperative telemedicine studies were larger-scale implementation studies that were primarily focused on utilizing pure telemedicine protocols through video consultations and telephone [4,18-20]. These studies reported surgical cancellation rates ranging from 1.3% to 2.95% [4,20], and one study even experienced surgical delays due to incomplete tele-preoperative assessments [18].

This study aimed to test a hybrid protocol that combines both physical examination and virtual assessment, involving history taking and risk assessment. Traditionally, in-person preoperative anesthesia assessments were conducted to allow for physical examination of the airway and cardiorespiratory system, as these were deemed necessary by anesthesiologists for satisfactory evaluations [21]. The reliability of virtual airway examinations has been a concern for anesthesiologists and a potential barrier to the widespread adoption of telemedicine for preoperative evaluations [22]. While our telemedicine protocol incorporates video conferencing capabilities to enhance visual assessment, challenges in assessing the Mallampati score were encountered due to varying lighting conditions and camera capabilities of user devices. Therefore, we incorporated the upper lip bite test as part of our virtual airway assessment during video consultations, as it has been shown to have a high predictive value for difficult airways [23]. Both the upper lip bite test and physical airway examination were performed to establish a correlation. It is worth noting that both assessments correlated well, with no reported incidents of difficult airways among the recruited patients. Additionally, our conservative inclusion criteria for both patients and surgeries were implemented to prioritize patient safety, minimize surgical cancellations, and gain stakeholder confidence. The hybrid protocol also resulted in time savings by eliminating wait times for physical consultations and test results. Pan A [24] demonstrated the extension of telemedicine to cardiac surgical patients, achieving a safety profile equivalent to physical consultations without any surgical cancellations or morbidity. This highlights the potential for video consultation to be expanded to selected ASA III patients with well-defined surgical criteria and buy-in from relevant stakeholders, such as patients, surgeons, and anesthesiologists.

Our survey revealed a high level of patient satisfaction, with 99% of respondents expressing satisfaction with the quality of video consultations and 88% indicating a preference for video consultations over physical consultations in the future. This finding aligns with the results of other anesthesia teleconsultation studies [7,11,19] and could be attributed to the conservative selection criteria and increased acceptance by patients during the pandemic. Wienhold et al. also reported high technical feasibility, medical feasibility, and patient satisfaction among their ASA I and II patients in their implementation study [19].

Despite these positive findings, our study has several limitations. Firstly, it is an observational study with a small sample size, and selection bias may exist as eligible patients who declined video consultations were not included. Secondly, although the survey response rate was high, there is a possibility of response bias as the views of non-responders were not captured. Thirdly, our hybrid protocol includes a physical examination component and may not reflect a fully virtual consultation. Notwithstanding these limitations, this feasibility study provides a foundation for the development of a basic protocol for the implementation of telemedicine in preoperative anesthesia evaluation. Future research can focus on further refining the medical and technical safety criteria to include more complex surgeries and patients, ultimately allowing telemedicine to progress alongside mainstream physical consultations and provide a reliable alternative for patients.

## Conclusions

Our pre-anesthetic video consultation hybrid protocol demonstrated both technical and medical feasibility, resulting in high patient satisfaction rates. This hybrid protocol, which incorporates conservative patient and surgical inclusion criteria, includes a physical examination component to enhance safety and reduce the likelihood of surgical case cancellations. The success of this protocol establishes a foundation for the future sustainability and scalability of telemedicine in pre-anesthetic evaluation, allowing for the inclusion of patients with more complex medical conditions and surgical criteria.

## Appendices

**Table 5:** Post video consultation questionnaire

Please indicate your response to the following statements: Strongly Agree (5) / Agree (4)/ Neutral (3)/ Disagree (2)/ Strongly disagree (1)	
The technical setup for video consultation was easy for me.	
I understood the various anaesthesia options that were explained to me over the video consultation.	
I understood the preoperative instructions regarding fasting and medications during the video consultation.	
I had time to ask questions during the video consultation.	
My concerns were adequately addressed during the video consultation.	
I found the duration of video consultation to be adequate.	
Overall, I am satisfied with the quality of service provided over the video consultation.	
I would choose video consultation over physical consultation for any future preoperative anaesthesia evaluation.	
Do you have any other feedback on how we can improve?	
